# Concerns of HIV-positive migrant workers in COVID-19 pandemic: A call for action

**DOI:** 10.7189/jogh.10.020342

**Published:** 2020-12

**Authors:** Ali Ahmed, Juman Dujaili, Anisha Kaur Sandhu, Furqan Khurshid Hashmi

**Affiliations:** 1 School of Pharmacy, Monash University, Bandar Sunway, Malaysia; 2 University College of Pharmacy, University of the Punjab, Lahore, Pakistan

The UNAIDS set a goal to eradicate the acquired immunodeficiency syndrome (AIDS) epidemic worldwide by 2030 [1]. The global coronavirus disease 2019 (COVID-19) pandemic is deemed to pose a greater risk to patients with chronic diseases, including those that are affected by human immunodeficiency virus HIV/AIDS [2]. During this time, it is crucial to properly identify HIV/AIDS patients to ensure that they continue to receive timely and equitable access to health care and health support as they are increasingly vulnerable to COVID-19 consequences [2].

COVID-19 emerged in China at the end of 2019 spreading rapidly to infect over 213 countries and territories worldwide resulting in more than 11 500 000 confirmed cases and 500 000 deaths as of 6th July 2020 [3]. While it has been realized that patients with chronic conditions require additional health support to mitigate risk of COVID-19, it is also important to recognise the vulnerability of chronically-ill migrant populations whose health issues are often neglected [2]. This is especially of concern in HIV/AIDS-infected migrant populations who avoid deportation and are considered illegal as immigration laws in various countries do not permit carriers of HIV infection to work or stay regardless of their legal status [4,5]. Reports have confirmed that Russia, Middle-East and South-East Asian countries usually deport migrant workers who test positive for HIV [6]. Reports have confirmed that until 2014, approximately 51 000 HIV-positive patients have been deported back to Pakistan from the Gulf countries [7]. In a bid to escape deportation, these HIV positive migrants are often forced to obtain antiretroviral therapy (ART) illegally through exploitation of informal channels, friends or family to continue treating their condition [6,8]. In other cases, these migrants are forced to buy expensively priced drugs (or in desperation, share the highly sought treatment with other migrants in order to afford it) [8].

Additionally, access to health care has been challenging during the COVID-19 pandemic with governments globally opting to curb the spread of the virus by introducing different national measures including lockdown or movement control, self-isolation and social distancing [2]. The lockdown and quarantine measures taken by most countries have been daunting for its HIV/AIDS-infected migrant population (legal or illegal) many of whom have been forced into unemployment and are unsure how to access appropriate health support, obtain essential medications or treatment [6]. Not only do these migrants face difficulties in accessing public and health services they may have depended on previously, but they also may be living in inadequate accommodation where they are unable to observe safe and appropriate social distancing and hygiene protocols [5].

Migrants living with HIV/AIDS often feel reluctant to inform their employers of their condition due to the social stigma associated with being a HIV/AIDS patient [4]. Hence, they continue to work, exposing themselves and others to risk [4]. Despite the increased risk of contracting COVID-19, HIV-infected migrants in the UK tend not to be eligible for financial employment assistance though they may be in unstable employment. Hence, they face increasing difficulties in earning or receiving sufficient funds to afford ART medication [4,9].

Ensuring a migrants’ good health is critical for the countries in which they originate from and move to [10] However, reports globally show that migrants are often subject to health inequalities due to lack of access to adequate health care as a result of neglect, exclusion, discrimination, unfavourable employment conditions causing ill health, inadequate living conditions, financial constraints or lack of appropriate legal status [10]. Studies conducted in Australia and Europe respectively show that migrants are behind in fulfilling the UNAIDS 90-90-90 target (a strategy where 90% of HIV patients should be aware of their HIV status, receive sustained antiretroviral therapy and achieve viral suppression by the year 2020) and more prone to being lost to follow-up ART treatment when compared to non-migrant population [11,12]. Access to health care is also affected by a divide in the skill level of employment that migrants are hired for [10]. Highly-skilled professional foreigners, often referred to as ‘expatriates’, tend to experience better health care access when compared to migrants working in low-skilled employment [10]. The pressures of social adjustment and poor health literacy also increase the migrant workers vulnerability to deteriorating health in times of a pandemic as they navigate through the difficulties of integrating into a foreign culture and language, while accepting the loss of their traditional values, beliefs and support systems [13]. Overall, these factors further expose a highly-vulnerable migrant population suffering from HIV/AIDS to the threat of contracting the COVID-19 virus [8].

**Figure:**
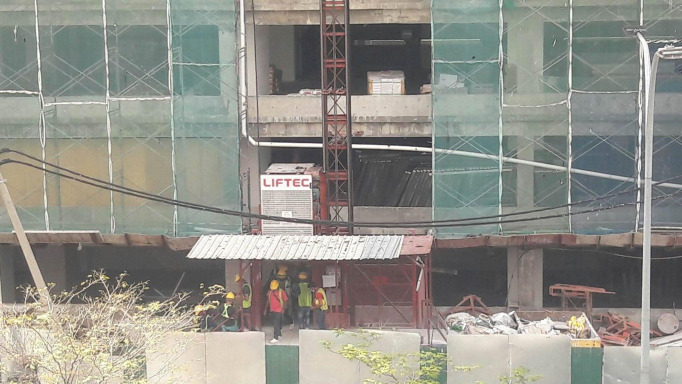
Photo: from the authors’ own collection, used with permission.

HIV/AIDS patients are advised to observe precautions similar to general recommendations for COVID-19 such as hand washing, cough and sneezing etiquettes, and social distancing [14]. They should also be assured with a 30-day supply of ART treatment as well as adequate supply of appropriate medications to treat other acute or chronic illnesses or addictions [14]. While sufficient evidence is not available at present to link HIV/AIDS patients undergoing ART treatment to a higher susceptibility of COVID-19 virus, nonetheless individuals with advanced-stage or poorly regulated HIV (low CD4 T-lymphocytes count and high viral load) are at increased risk of infection, health complications and therefore, stand an increased chance of contracting COVID-19 [14].

Studies have shown that migrants seldom bring infections that pose a threat to the population of the host country [13]. In the past, outbreaks of hepatitis A, B, HIV and tuberculosis have occurred in non-endemic regions due to a lack of timely treatment provision to refugees [13]. A study conducted by Mendelsohn et al. in Malaysia compared the adherence of ART between refugees and the host population. It has been noted that, if asylum seekers are included in primary care, the high levels of compliance achieved (equal to those in the domestic population) have decreased onward HIV transmission [15].

## SUMMARY AND CONCLUSION

In short, most migrants (whether legal or illegal) lack adequate health entitlements and face health inequalities that prevent them from accessing health care in a safe, appropriate and fair manner [13]. Some countries, such as Thailand and Spain have ensured equal access to health care for all legal and illegal migrants in accordance with the human rights framework [13]. The World Health Organization (WHO) should provide guidelines to all countries with HIV/AIDS infected migrants (whether legal or illegal) to adhere to so the migrant population continues to receive fair, assured and uninterrupted supply of ART treatment during the COVID-19 pandemic to maintain their immunity, health and decrease risk of COVID-19 contraction. This will ensure another step is taken in a positive direction for the WHO to fulfil its slogan of “Health for all”.
